# Growth-uncoupled isoprenoid synthesis in *Rhodobacter sphaeroides*

**DOI:** 10.1186/s13068-020-01765-1

**Published:** 2020-07-13

**Authors:** Enrico Orsi, Ioannis Mougiakos, Wilbert Post, Jules Beekwilder, Marco Dompè, Gerrit Eggink, John van der Oost, Servé W. M. Kengen, Ruud A. Weusthuis

**Affiliations:** 1 Bioprocess Engineering, Wageningen University, Droevendaalsesteeg 1, 6708 PB Wageningen, The Netherlands; 2 Laboratory of Microbiology, Wageningen University, Stippeneng 4, 6708 WE Wageningen, The Netherlands; 3 Wageningen Plant Research, 6700AA Wageningen, The Netherlands; 4 Physical Chemistry and Soft Matter, Wageningen University, Stippeneng 4, 6708 WE Wageningen, The Netherlands; 5 Wageningen Food & Biobased Research, 6708WG Wageningen, The Netherlands; 6 Present Address: Systems and Synthetic Metabolism Group, Max Planck Institute of Molecular Plant Physiology, Am Mühlenberg 1, 14476 Potsdam, Germany; 7 Present Address: Helmholtz Institute for RNA-based Infection Research (HIRI), Helmholtz-Centre for Infection Research (HZI), 97080 Würzburg, Germany

**Keywords:** *Rhodobacter sphaeroides*, Isoprenoid biosynthesis, PHB, MEP, MVA, Growth-independent production

## Abstract

**Background:**

Microbial cell factories are usually engineered and employed for cultivations that combine product synthesis with growth. Such a strategy inevitably invests part of the substrate pool towards the generation of biomass and cellular maintenance. Hence, engineering strains for the formation of a specific product under non-growth conditions would allow to reach higher product yields. In this respect, isoprenoid biosynthesis represents an extensively studied example of growth-coupled synthesis with rather unexplored potential for growth-independent production. *Rhodobacter sphaeroides* is a model bacterium for isoprenoid biosynthesis, either via the native 2-methyl-d-erythritol 4-phosphate (MEP) pathway or the heterologous mevalonate (MVA) pathway, and for poly-β-hydroxybutyrate (PHB) biosynthesis.

**Results:**

This study investigates the use of this bacterium for growth-independent production of isoprenoids, with amorpha-4,11-diene as reporter molecule. For this purpose, we employed the recently developed Cas9-based genome editing tool for *R. sphaeroides* to rapidly construct single and double deletion mutant strains of the MEP and PHB pathways, and we subsequently transformed the strains with the amorphadiene producing plasmid. Furthermore, we employed ^13^C-metabolic flux ratio analysis to monitor the changes in the isoprenoid metabolic fluxes under different cultivation conditions. We demonstrated that active flux via both isoprenoid pathways while inactivating PHB synthesis maximizes growth-coupled isoprenoid synthesis. On the other hand, the strain that showed the highest growth-independent isoprenoid yield and productivity, combined the plasmid-based heterologous expression of the orthogonal MVA pathway with the inactivation of the native MEP and PHB production pathways.

**Conclusions:**

Apart from proposing a microbial cell factory for growth-independent isoprenoid synthesis, this work provides novel insights about the interaction of MEP and MVA pathways under different growth conditions.

## Background

**Fig. 1:**
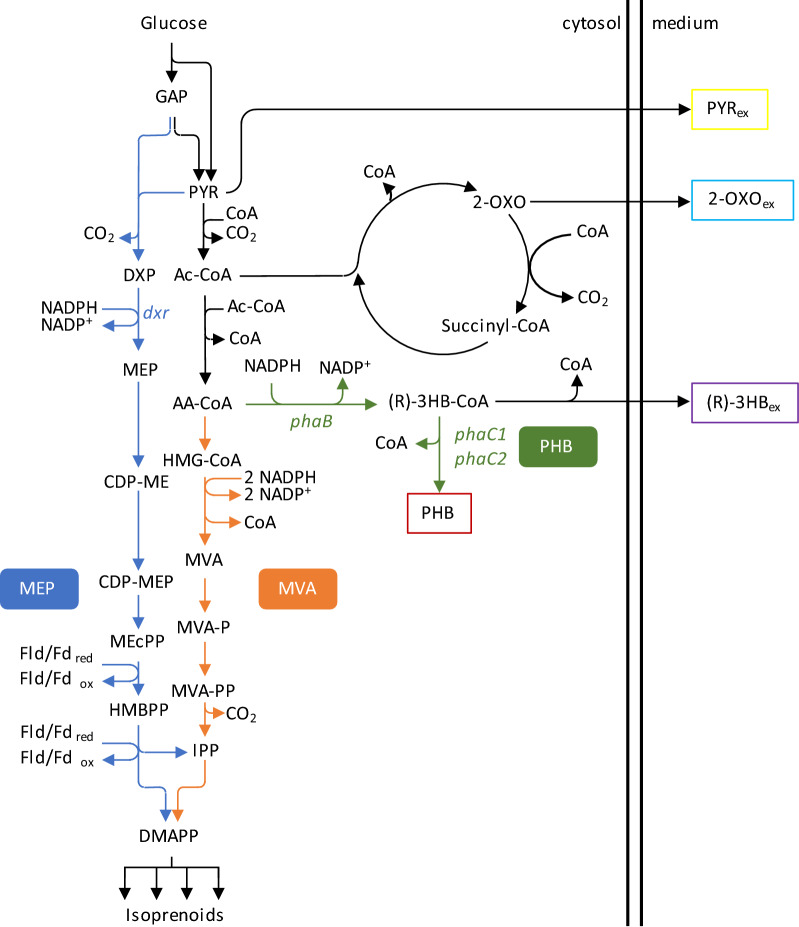
Network investigated in this work. The metabolic map shows the steps involved in the biochemical conversion of glucose to isoprenoids. Isoprenoid biosynthesis can occur via the two orthogonal 2-C-methyl-d-erythritol 4-phosphate (MEP, blue arrows) and mevalonate (MVA, orange arrows) pathways. For the MEP pathway, the 1-deoxy-d-xylulose 5-phosphate reductoisomerase (*dxr*) gene is shown. This gene is targeted for inactivating the endogenous isoprenoid pathway. Both modules branch from the central metabolism and converge to isopentenyl-diphosphate (IPP) and dimethylallyl-diphosphate (DMAPP), which are the precursors of all isoprenoids. Moreover, the biosynthetic pathway of the storage compound poly-β-hydroxybutyrate (PHB) is included (box with red outline). This consists of two enzymatic reactions encoded by the genes *phaB* and *phaC1* and *phaC2* (both in green). The network includes also the schematic representation of the Krebs cycle, which includes conversion of 2-oxoglutarate (2-OXO) to succinyl-CoA. Eventually, accumulation of PYR or 2-OXO can result in their secretion in the medium (boxes with yellow and light-blue outline, respectively). Additionally, conversion of (R)-3hydroxybturyryl-CoA ((R)-3HB-CoA) to (R)-3HB can result in the secretion of the latter compound (box with purple outline). Other abbreviations: reduced flavodoxin (Fld), ferredoxin (Fd), red: reduced, ox: oxidized. GAP (glyceraldehyde-3-phosphate), PYR (pyruvate), CDP-ME (4-(cytidine 5′-diphospho)-2-C-methyl-d-erythritol), CDP-MEP (2-phospho-4-(cytidine 5′-diphospho)-2-C-methyl-d-erythritol), MEcPP (2-C-methyl-d-erythritol 2,4-cyclodiphosphate), Ac-CoA (acetyl-CoA), AA-CoA (acetoacetyl-CoA), HMG-CoA (S)-3-hydroxy-3-methylglutaryl-CoA), MVA-P ((R)-5-phosphomevalonate), MVA-PP ((R)-5-diphosphomevalonate)

Isoprenoids (also known as terpenoids) have great industrial value as ingredients of pharmaceuticals, perfumes, food flavourings and most recently biofuels [1–6]. They are formed by the condensation of the five-carbon monomers isopentenyl pyrophosphate (IPP) and its isomer dimethylallyl pyrophosphate (DMAPP). The two naturally existing IPP/DMAPP production pathways are the 2-C-methylerythritol 4-phosphate (MEP) pathway and the mevalonate (MVA) pathway [7]. While the former branches in the central metabolism from glyceraldehyde 3-phosphate and pyruvate, the latter uses acetoacetyl-CoA (AA-CoA) as precursor (Fig. 1). These two pathways are, with few exceptions, phylogenetically distinct: the MEP pathway is present in prokaryotes, while the MVA pathway is found in archaea and eukaryotes [8]. Plants express both metabolic routes, with the MEP pathway compartmentalized in the chloroplasts and the MVA pathway expressed in the cytosol [9]. Since the first implementation of a heterologous MVA pathway in *Escherichia coli* [10], the number of studies focusing on the synthesis of isoprenoids via microbial cell factories has increased a lot. Efforts for improving bioproduction have been focusing either on engineering of the endogenous MEP pathway [11–13], or by co-expressing the heterologous MVA counterpart [10, 14, 15]. Moreover, to limit the effect of unfavourable regulatory control of the endogenous pathway, substitution by an orthogonal isoprenoid pathway has been reported [16].

In microorganisms, isoprenoids are often membrane-bound molecules—like carotenoids, ubiquinones, chlorophylls and sterols—which are indispensable for growth [17, 18]. Therefore, during a batch cultivation, their volumetric concentration is expected to increase as consequence of microbial growth inside the reactor. Such type of metabolism for isoprenoid biosynthesis is defined as growth-coupled. For strain improvement purposes, growth-coupled production has been largely employed. In such a scenario, the product of interest becomes a mandatory by-product of growth, and therefore microbial growth becomes the driving force of production [19]. This production–growth association has already been exploited for enhancing isoprenoid biosynthesis by laboratory evolution [20].

Nevertheless, growth-coupled production contains an inherent trade-off between substrate use for (i) biomass production and maintenance, and (ii) product formation [21]. Thus, if biomass formation is prevented, in principle more substrate is available for product synthesis.

Microbial metabolism has been engineered to produce non-native isoprenoid molecules as pharmaceuticals, perfumes, food flavourings and biofuels [1–6]. These compounds are not required for growth, and often excreted. Coupling of product formation to microbial growth is therefore not a necessity, and growth-uncoupled production would be an advantageous option.

Apart from C, H and O, isoprenoids do not contain other biomass-specific elements like P, S or N. Therefore, under P, S or N-limited incubation conditions, glucose can in principle be converted into isoprenoid but not into microbial biomass. Improved isoprenoid/biomass ratios have already been obtained by using nutrient-limited culturing conditions [22–25]. Nonetheless, as a highly regulated primary metabolism, isoprenoid biosynthesis has been largely optimized for growth-coupled production, and decoupling isoprenoid synthesis from microbial growth has been overlooked as a production strategy.

In a recent study, the concept of redesigning biosynthetic networks based on orthogonality principles was introduced [26]. This idea entails that a non-native metabolic route that minimizes the interaction with the endogenous biomass-producing pathways can be exploited for bioproduction. Intrinsic independence exists between the two isoprenoid pathways MEP and MVA. Therefore, regulatory control affecting the MEP pathway should not affect the MVA pathway, and vice versa. Moreover, in a recent study, functional replacement of the native MEP with the heterologous MVA pathway was described in the bacterium *Rhodobacter sphaeroides* [16], a microbial platform organism that is gaining interest for isoprenoid biosynthesis. This organism is able to synthesize intracellular membranes which can accommodate isoprenoids such as carotenoids and bacteriochlorophylls. Moreover, it is also a natural producer of coenzyme Q_10_. Apart from these native isoprenoid molecules, heterologous production of lycopene [27] and sesquiterpenes [28] has also been reported in this species.

Growth and isoprenoid synthesis in *R. sphaeroides* has been studied using defined medium, where the introduction of the heterologous MVA pathway revealed potential for growth-independent isoprenoid biosynthesis [29]. Under these conditions, the storage compound poly-β-hydroxybutyrate (PHB) was also accumulated [29]. Additionally, a mutual stimulating effect between the MEP and MVA pathways has been observed [30].

In this study, we investigate the behaviour of the two isoprenoid pathways for amorphadiene production in *R. sphaeroides* during different growth modes, as well as their interaction with the pathway for the carbon- and energy reserve material poly-β-hydroxybutyrate, PHB. By means of ^13^C metabolic flux ratio analysis, we assess the effect of genetic modifications (i.e. for elimination of PHB accumulation) and environmental changes (nitrogen limitation) on isoprenoid pathways capacities. Ultimately, we demonstrate that exclusive use of the orthogonal MVA pathway in combination with elimination of PHB synthesis is a promising strategy for attaining growth-independent production of isoprenoids.

## Materials and methods

### Strains and standard cultivation conditions

The strains and plasmids used in this study are listed in Tables 1 and 2, respectively. The Rs265_*∆phaC1∆phaC2* strain was kindly donated by Isobionics BV. Preculturing of *R. sphaeroides* was performed in 250-mL Erlenmeyer flasks containing 25 mL of modified Sistrom’s minimal medium (SMM). As previously described [30], the medium contained glucose (3.0 g/L) as carbon source, and NH_4_Cl (1.0 g/L) as nitrogen source. Moreover, the SMM contained (per litre): 3.48 g KH_2_PO_4_, 0.1 g glutamic acid, 0.04 g l-aspartic acid, 0.5 g NaCl, 0.02 g nitrilotriacetic acid, 0.3 g MgSO_4_·7H_2_O, 0.0334 g CaCl_2_·2H_2_O, 0.002 g FeSO_4_·7H_2_O, and 0.0002 g (NH_4_)_6_Mo_7_O_24_. Trace elements were added (0.01% v/v) from a stock solution containing: 17.65 g/L disodium EDTA, 109.5 g/L ZnSO_4_·7H_2_O, 50 g/L FeSO_4_·7H_2_O, 15.4 g/L MnSO_4_·7H_2_O, 3.92 g/L CuSO_4_·5H_2_O, 2.48 g/L Co(NO_3_)_2_·6H_2_O, and 0.0114 g/L H_3_BO_3_. Vitamins were added (0.01% v/v) from a stock containing: 10 g/L nicotinic acid, 5 g/L thiamine HCl, and 0.1 g/L biotin.

**Table 1:** Strains used in this study

Strain	Description	Source of reference
*E. coli*		
S17-1	Host strain for conjugation, *thi pro recA hsdR* [RP4–2Tc::Mu–Km::Tn7] *Tp*^*r*^*Sm*^*r*^	Laboratory stock
*R. sphaeroides*		
Rs265	Wild-type	Derivative of ATCC35035, Isobionics BV
Rs265_*∆phaC1∆phaC2*	Rs265 with the PHB biosynthetic pathway knocked-out via *phaC1* and *phaC2* deletion	Isobionics BV
Rs265_*∆phaB*	Rs265 with the PHB biosynthetic pathway knocked-out via *phaB* deletion	[31]
Rs265-MVA_*∆dxr*	Rs265 with chromosomally integrated the MVA pathway operon and knocked-out the endogenous MEP pathway via *dxr* deletion	[16]
Rs265-MVA_*∆dxr∆phaB*	Rs265 with chromosomally integrated the MVA pathway operon and knocked-out the endogenous MEP (via *dxr* deletion) and PHB (via *phaB* deletion) biosynthetic pathways	This study

**Table 2:** Plasmids used in this study

Plasmid	Description	Source of reference
pBBR-*ads*	pBBR1MCS-2 + *crtE* promoter and *ads* (amorphadiene synthase)	[29]
pBBR-MVA-*ads*	pBBR1MCS-2 + *crtE* promoter controlling MVA enzymes and *ads*	[28]
pBBR_ Cas9_*ΔphaB*_HR	pBBR1MCS-2 + codon harmonized *cas9* sequence, sgRNA targeting *phaB* and 1 kb homologous-recombination flanks for recombination with *phaB*	[31]

### Generation of double-KO strain via CRISPR–Cas9 counter-selection

The primers used in this study are listed in Additional file 1: Table S1. Deletion of *phaB* was performed as previously described [31], using the pBBR_Cas9_*∆phaB*_HR plasmid. Such plasmid was transferred from *E. coli* S17 cells to Rs265-MVA_*∆dxr* via diparental conjugation, resulting in the double mutant strain Rs265-MVA_*∆dxr∆phaB*. By employing the pBBR_Cas9_*∆phaB*_HR plasmid, *phaB* could be removed by homologous-recombination, and Cas9-based counter-selection of cells with intact genomic copies of *phaB*.

### Diparental conjugation of *R. sphaeroides*

Diparental conjugation for transferring amorphadiene producing plasmids was performed as previously described [30] using RÄ medium. Such medium contained, per litre: 3 g malic acid, 0.2 g MgSO_4_·7H_2_O, 1.2 g (NH_4_)_2_SO_4_, 0.07 g CaCl_2_·2H_2_O, 1.5 mL of microelements stock solution, 2 mL of vitamin stock solution and 5 mL of phosphate buffer. In case of RÄ agar medium, 15 g/L agar was added. The microelements solution contained: 0.5 g/L Fe(II)-Citrate, 0.02 g/L MnCl_2_·4H_2_O, 0.005 g/L ZnCl_2_, 0.0025 g/L KBr, 0.0025 g/L KI, 0.0023 g/L CuSO_4_·5H_2_O, 0.041 g/L Na_2_MoO, 0.005 g/L CoCl_2_·6H_2_O, 0.0005 g/L SnCl_2_·2H_2_O, 0.0006 g/L BaCl_2_·2H_2_O, 0.031 g/L AlCl, 0.41 g/L H_3_BO_3_, 0.02 g/L EDTA. The vitamin solution contained: 0.2 g/L nicotinic acid, 0.4 g/L thiamine HCl, 0.008 g/L biotin, 0.2 g/L nicotinamide. The phosphate buffer contained 0.6 g/L KH_2_PO_4_ and 0.9 g/L K_2_HPO_4_.

### Cultivation in nitrogen excess and nitrogen-limited conditions

After overnight preculturing on SMM, *R. sphaeroides* cultures were transferred to fresh SMM with a starting OD_600_ of 0.1, and incubated at 30 °C with 250 rpm. Cultivations were performed for biological triplicates in 250-mL Erlenmeyer flasks, each filled with 45 mL of SMM medium and 5 mL of filter-sterilized dodecane. SMM composition differed between nitrogen excess condition and nitrogen-limited conditions only in the initial NH_4_Cl concentration: 1.0 g/L and 0.25 g/L, respectively. Initial glucose concentration remained 3.0 g/L in both cases. Amorphadiene titers were measured after glucose depletion. This occurred after 24 h (for nitrogen excess condition) or after 48 h (for nitrogen-limited condition). At the same time, the content of the flasks was harvested by centrifugation and further processed for analytical measurements.

### ^13^C-metabolic flux ratio analysis of isoprenoid biosynthesis

Isoprenoid flux ratios analyses were performed as previously described [30] in 10-mL Erlenmeyer flasks containing 1.8 mL of labelled SMM medium and 0.2 mL of filter-sterilized dodecane. [1-^13^C]- and [4-^13^C]-glucose tracers had an initial concentration of 3.0 g/L. For nitrogen excess and limited conditions, the initial NH_4_Cl concentration used was the same as for the cultivations in 250-mL flasks: 1.0 and 0.25 g/L, respectively. Samples for GC–MS measurement were taken at glucose depletion. MEP and MVA pathways capacities were obtained by multiplying the flux ratios determined via GC–MS with the amorphadiene titers measured via GC-FID for the 250-mL cultivations.

### Cultivation under resting cell conditions

For resting cell cultivations, exponentially growing cells on SMM with nitrogen excess conditions were incubated until mid-exponential phase. Then, cells were pelleted by centrifugation at 4255 g for 10 min at room temperature. Subsequently, pellets were washed twice with sterile physiologic solution (NaCl 9 g/L) and centrifuged at 4255*g* for 5 min. Washed pellets were inoculated with a starting OD_600_ of 1.0 on ‘nitrogen-free SMM’ containing 5.0 g/L glucose and 0.0 g/L NH_4_Cl. Initial biomass concentration was determined at the moment of the inoculum with a sample of 5 mL for TOC-L analysis. Then, incubation proceeded at 30 °C with 250 rpm. Samples for OD_600_, pH and amorphadiene measurements were taken at a regular interval until the pH dropped below 5.5. Determination of productivity and yields were performed for samples taken within the first 24 h.

### Analytics

Cell density was monitored by measuring the optical density at 600 nm (OD_600_). Amorphadiene concentration was measured via GC-FID as previously described [29]. Glucose, PHB and organic acid concentrations were determined via (U)HPLC as previously described [29]. For pyruvate and crotonic acid (resulting from PHB hydrolysis) identification, DAD detector was used, while for glucose, 2-oxoglutarate and 3-hydroxybutyrate determination, the RID detector was used. Determination of biomass concentration via TOC-L was performed by measuring the nitrogen content of the pellet, which was then used to calculate the active biomass concentration using the elemental composition of *R. sphaeroides* of CH_1.99_O_0.5_N_0.19_ [29]. Identification of unknown compounds in the spent medium was obtained via ^1^H-nuclear magnetic resonance spectroscopy (^1^H-NMR) measurements performed in D_2_O on a Bruker Avance III 400 MHz NMR spectrometer.

## Results

### Prevention of PHB formation and its effect on amorphadiene biosynthesis

**Fig. 2:**
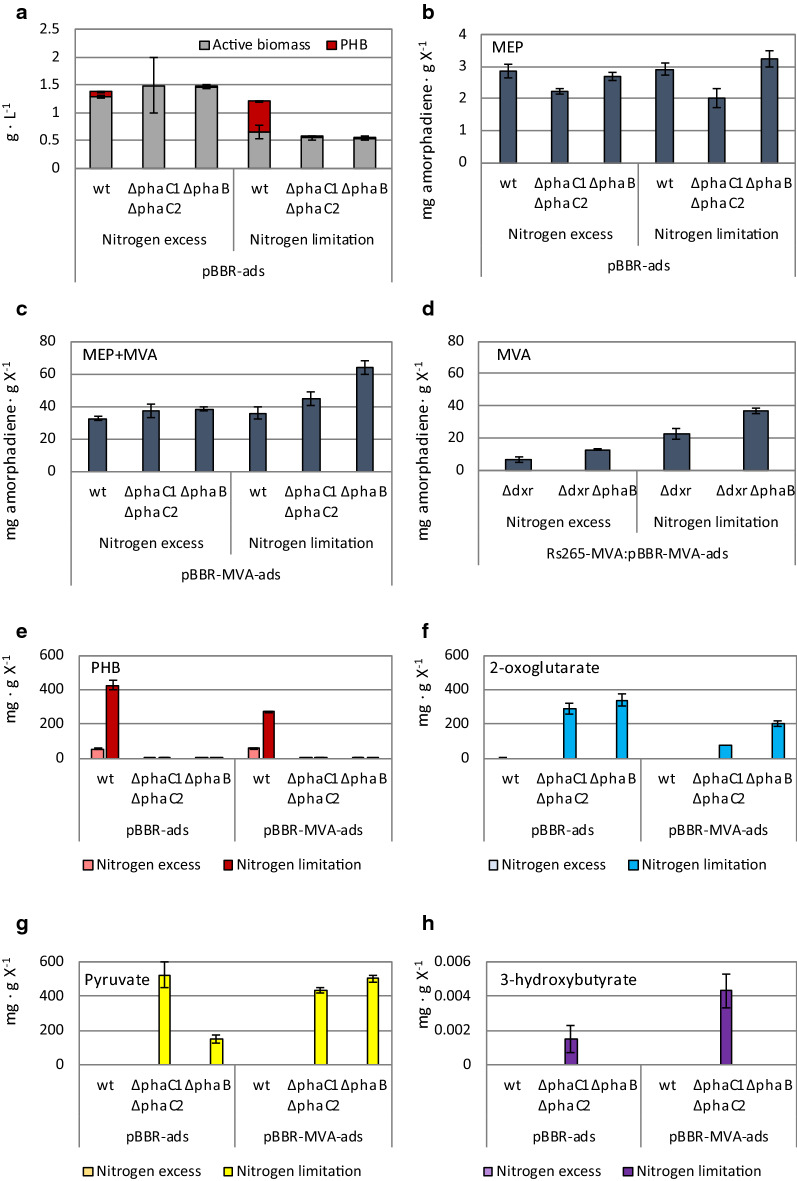
Deletion of PHB pathway and effect on amorphadiene yield on biomass. **a** Effect of initial medium C/N ratio on active biomass and PHB concentrations. **b**–**d** mg amorphadiene g of biomass^−1^ for wt, *∆phaC1∆phaC2* and *∆phaB* background in strains relying on **b** only MEP pathway (pBBR-*ads*), **c** MEP and MVA pathway (pBBR-MVA-*ads*), **d** only MVA pathway (Rs265-MVA:pBBR-MVA-*ads* strain). **e**, **f** mg g of biomass^−1^ calculated for **e** PHB, **f** 2-oxoglutarate, **g** pyruvate and **h** 3-hydroxybutyrate during nitrogen excess and nitrogen limited conditions

Culturing *R. sphaeroides* under nitrogen-limited conditions could theoretically result in growth-independent isoprenoid synthesis via the native MEP and the heterologous MVA pathways. However, upon consumption of the limited available nitrogen, *R. sphaeroides* stores excess carbon intracellularly as PHB, a nitrogen-free carbon and energy storage compound [29]. Aiming to increase isoprenoid production, we reasoned that deletion of one of the PHB synthesis genes would block PHB production under nitrogen-limited conditions and could therefore increase the flux through the MVA pathway. Deletion of the *phaC1* and *phaC2* genes, that code for the PHB polymerase, is the established approach for eliminating PHB biosynthesis in *R. sphaeroides* [32–35]. Nonetheless, this does not prevent activity of the NADPH-dependent acetoacetyl-CoA reductase PhaB, which could result in the undesired accumulation of 3-hydroxybutyryl-CoA or excretion of 3-hydroxybutyrate (Fig. 1). In a recent study, we demonstrated that deletion of the *phaB* gene prevents PHB biosynthesis [31]. Here, we confirmed that the deletion of either the *phaB* gene (Rs265_*∆phaB* strain) or the combined deletion of the *phaC1* and *phaC2* genes (Rs265_*∆phaC1∆phaC2* strain) prevents PHB formation both under nitrogen excess and nitrogen-limited conditions (Fig. 2a). As observed before [29], the wild-type (Rs265) strain produced substantial amounts of PHB, especially under nitrogen-limiting conditions.

The pBBR-*ads* plasmid harbouring the heterologous amorphadiene synthase gene was transferred to the various *R. sphaeroides* strains by conjugation. The resulting strains were cultured under both nitrogen excess and nitrogen-limited conditions. At glucose depletion, we determined (Table 3): amorphadiene titers (*C*_*P*_), yield of amorphadiene on glucose (*Y*_*P/S*_), active biomass concentration (*C*_*X*_) and the amorphadiene on biomass ratio (*Y*_*P/X*_). We observed that, for the Rs265_*∆phaB*:pBBR-*ads* and Rs265_*∆phaC1∆phaC2*:pBBR-*ads* strains that use only the endogenous MEP pathway (MEP-only strains), elimination of PHB synthesis does not result in higher amorphadiene/biomass ratios compared to the Rs265 *wt* strain (Fig. 2b, Table 3). In fact, these ratios remained unaffected when moving from nitrogen excess to nitrogen-limited conditions (Table 3) at a value of 2.9 ± 0.2 mg · g of biomass^−1^. Interestingly, the Rs265*_∆phaC1∆phaC2*:pBBR-*ads* strain showed an even lower amorphadiene/biomass ratio compared to the Rs265:pBBR-*ads* and the Rs265*_∆phaB*:pBBR-*ads* strains (Table 3). In summary, when only the endogenous MEP pathway was active, isoprenoid biosynthesis appeared to be strictly growth-coupled. Moreover, the MEP flux was insensitive or negatively affected by the impaired PHB synthesis.

**Table 3:** Titers and yields obtained from shaking flasks experiments of strains Rs265 and Rs265-MVA

Plasmid	Condition	Genomic background	Amorphadiene titers, *C*_*P*_ (mg · L^−1^)		Amorphadiene yield on glucose, *Y*_*P/S*_ (mg · g^−1^)		Final Biomass concentration, *C*_*X*_ (g · L^−1^)		Amorphadiene on Biomass, *Y*_*P/X*_ (mg · g^−1^)	
			Value	St. dev.	Value	St. dev.	Value	St. dev.	Value	St. dev.
pBBR-ads	Nitrogen excess	*wt*	4.4	0.3	1.41	0.15	1.533	0.062	2.9	0.2
		*ΔphaC1 ΔphaC2*	3.3	0.1	ND	ND	1.491	0.000	2.2	0.1
		*ΔphaB*	4.7	0.2	1.49	0.07	1.724	0.110	2.7	0.1
	Nitrogen limited	*wt*	2.4	0.2	0.73	0.05	0.805	0.122	2.9	0.2
		*ΔphaC1 ΔphaC2*	1.4	0.2	ND.	ND.	0.693	0.034	2.0	0.3
		*ΔphaB*	2.2	0.2	0.69	0.04	0.693	0.112	3.2	0.2
pBBR-MVA-ads	Nitrogen excess	*wt*	41.8	1.8	13.64	0.56	1.289	0.019	32.4	1.4
		*ΔphaC1 ΔphaC2*	39.3	4.4	ND.	ND.	1.060	0.856	37.1	4.2
		*ΔphaB*	56.4	2.1	18.25	0.68	1.466	0.036	38.5	1.5
	Nitrogen limited	*wt*	23.6	2.3	7.65	0.76	0.656	0.115	35.9	3.6
		*ΔphaC1 ΔphaC2*	24.1	2.1	ND.	ND.	0.539	0.260	44.8	3.9
		*ΔphaB*	34.4	2.2	11.38	0.73	0.539	0.022	63.7	4.0
pBBR-MVA-ads	Nitrogen excess	*MVAint_Δdxr*	15.6	3.9	3.41	0.23	2.229	0.144	7.0	1.7
		*MVAint_ΔdxrΔphaB*	28.0	1.0	9.67	0.86	2.187	0.080	12.8	0.5
	Nitrogen limited	*MVAint_Δdxr*	13.5	1.9	3.80	0.82	0.597	0.142	22.6	3.1
		*MVAint_ΔdxrΔphaB*	19.5	0.8	5.65	0.29	0.528	0.161	36.9	1.6

Data were obtained from end-point measurements under nitrogen excess and nitrogen-limited conditions

*ND* not determined

We subsequently transformed the available Rs265, Rs265*_∆phaC1∆phaC2* and Rs265*_∆phaB* strains with the orthogonal MVA pathway, cloned in the pBBR-MVA-*ads* plasmid. The amorphadiene/biomass ratio increased 10- to 20-fold for all the strains tested (Fig. 2c, Table 3). The highest increase was observed for the *∆phaB* strain (Rs265*_∆phaB*:pBBR-MVA-*ads)*, reaching a ratio of 63.7 ± 4.0 mg · g of biomass^−1^ (Fig. 2c, Table 3). This value was significantly higher than the ratio reached by the strain with a functional PHB synthesis (Rs265:pBBR-MVA-*ads)*, which was 35.9 ± 3.6 mg · g of biomass^−1^ (Fig. 2c, Table 3).

Increase of the MVA pathway flux, as consequence of the *phaB* deletion, was confirmed also for the Rs265-MVA_*∆dxr* strain, for which the MEP pathway is inactivated via the deletion of the 1-deoxy-d-xylulose 5-phosphate reductoisomerase (*dxr*) gene, after genomic integration of the MVA pathway (Fig. 1). This strain relies exclusively on the non-native isoprenoid route (MVA-only) [16]. Also here, a substantial increase in the amorphadiene/biomass ratio was observed during both nitrogen excess and nitrogen-limited conditions (Fig. 2d, Table 3). The highest value observed was for the Rs265-MVA_*∆dxr∆phaB*:pBBR-MVA-*ads* strain, with 36.9 ± 1.6 mg · g of biomass^−1^ during nitrogen limitation.

In summary, although both the *∆phaC1∆phaC2* and *∆phaB* knockouts were equally effective in reducing PHB synthesis, the *∆phaB* knockout strain produced more amorphadiene, both volumetrically and per biomass unit (Table 3).

### Organic acids secretion as consequence of PHB deletion

We reasoned that comparing the secretion profiles between ∆*phaB* and ∆*phaC1*∆*phaC2* could provide additional insights on the beneficial effect of ∆*phaB* on isoprenoid synthesis. Therefore, we quantified by HLPC analysis the organic acids in the spent medium of Rs265, Rs265_∆*phaC1*∆*phaC2* and Rs265_∆*phaB* harbouring either pBBR-*ads* or pBBR-MVA-*ads* plasmids (Fig. 2e–h). For both ∆*phaB* and ∆*phaC1*∆*phaC2* strains, 2-oxoglutarate (80 to 340 mg · g of biomass^−1^, Fig. 2f) and pyruvate (150 to 500 mg · g of biomass^−1^, Fig. 2g) were the main by-products. Both compounds require coenzyme-A (CoA) for proceeding further in the metabolism via oxidative decarboxylation (Fig. 1). Excretion of these compounds suggests that free CoA is limiting when PHB biosynthesis is prevented.

The ∆*phaC1*∆*phaC2* strains secreted an additional unknown compound, which was identified as 3-hydroxybutyrate (3HB) by NMR (Additional file 1: Fig. S1). The spent medium for the Rs265_∆*phaC1*∆*phaC2*:pBBR-*ads* strain showed a value of 0.002 ± 0.001 mg 3HB · g of biomass^−1^ (Fig. 2h). In contrast, a value of 0.004 ± 0.001 mg 3HB · g of biomass^−1^ was observed for the Rs265_∆*phaC1*∆*phaC2*:pBBR-MVA-*ads* strain, which expressed the heterologous MVA pathway (Fig. 2h). Therefore, under nitrogen limitation and upon expression of the MVA pathway, the amount of 3HB secreted increased significantly compared to when only the MEP pathway was active.

### ^13^C metabolic flux ratio analysis of isoprenoid biosynthesis under different growth conditions

The Rs265_*∆phaB*:pBBR-MVA-*ads* strain, that overexpresses the MVA pathway and still has an active MEP pathway, showed the highest amorphadiene/biomass ratio under nitrogen-limited conditions (Fig. 2c, Table 3). Additionally, we observed that, independent from the cultivation conditions and the presence of an active PHB synthesis pathway, the dual-pathway (co-expressing MEP and MVA pathways) strains largely outperformed the single-pathway strains (Fig. 2b–d, Table 3). We therefore decided to further investigate the separate and combined contribution of the isoprenoid pathways to the amorphadiene production by ^13^C flux ratio analysis.

**Fig. 3:**
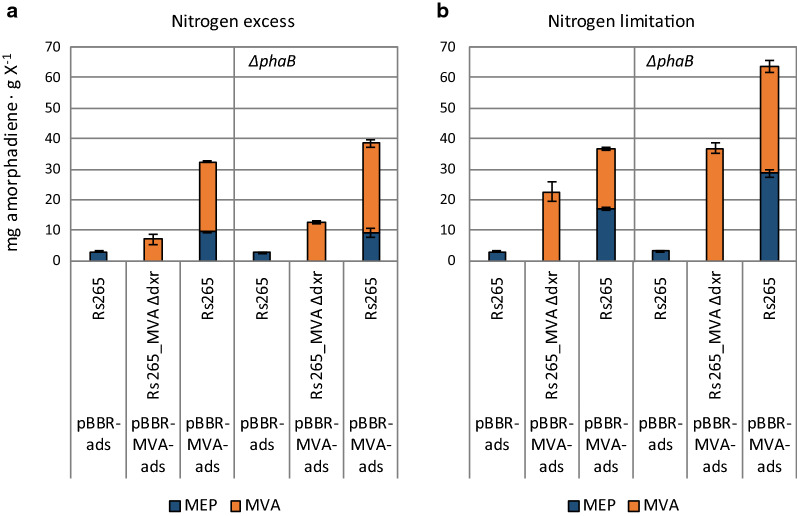
^13^C metabolic flux ratio analysis of *R. sphaeroides* with different initial C/N in the medium. The values are obtained from parallel labelling cultivation experiment. The isoprenoid flux ratios obtained were multiplied for the yield on biomass obtained for some of the strains of Fig. 2. **a** Nitrogen excess conditions: enough nitrogen is provided to support cell division until depletion of glucose. **b** Nitrogen limitation: nitrogen will be depleted from the medium before glucose, and cell division is expected to stop and allow PHB accumulation

MEP and MVA pathways are known to exert a reciprocal stimulation [30, 36]. To better understand their mode of interaction under the conditions tested, we determined their contribution via ^13^C metabolic flux ratio analysis of the Rs265:pBBR-MVA-*ads* and Rs265_*∆phaB*:pBBR-MVA-*ads* strains. We therefore compared the resulting amorphadiene/biomass ratios for each pathway with the ones determined for (i) the Rs265:pBBR-*ads* and Rs265_*∆phaB*:pBBR-*ads* (MEP-only) strains, (ii) the Rs265-MVA_*∆dxr*:pBBR-MVA-*ads* and (iii) Rs265-MVA_*∆dxr∆phaB*:pBBR-MVA-*ads* (MVA-only) strains. Previously, this ^13^C-method provided important insights on the flux ratios upon co-expression of the MEP and MVA pathways [30]. Under nitrogen excess condition, we observed that the dual-pathway strains with active MEP and MVA pathways showed a higher amorphadiene/biomass ratio for each isoprenoid route compared to when these were active individually (Fig. 3a). We therefore confirmed that, during nitrogen excess conditions, co-expression of the two isoprenoid pathways resulted in enhancement of their capacities in the Rs265 and Rs265_*∆phaB* strains harbouring the pBBR-MVA-*ads* plasmid (Fig. 3a, Table 4). Moreover, the capacity of the MVA pathway in the Rs265_*∆phaB* and Rs265-MVA_*∆dxr∆phaB* strains was even further enhanced by the *phaB* deletion, as made obvious by comparison to strains that still contain the *phaB* gene (Fig. 3a, Table 4). In contrast, the flux through the native MEP pathway remained unaffected by the *phaB* deletion. Thus, under nitrogen excess conditions, deletion of the *phaB* gene results in an increase of the isoprenoid flux exclusively via the MVA pathway (Table 4).

**Table 4:** Isoprenoid flux ratio calculated for the Rs265 and Rs265_*∆phaB* strains harbouring the pBBR-MVA-*ads* plasmid after growth on different initial nitrogen concentrations

Genomic background	Isoprenoid flux ratios				Pathway capacities (mg g biomass^−1^)			
	Nitrogen excess		Nitrogen limitation		Nitrogen excess		Nitrogen limitation	
	MEP	MVA	MEP	MVA	MEP	MVA	MEP	MVA
Rs265	0.29 ± 0.01	0.71 ± 0.01	0.46 ± 0.01	0.54 ± 0.1	9.4 ± 0.3	23.0 ± 0.3	16.9 ± 0.4	19.9 ± 0.5
Rs265_*∆phaB*	0.24 ± 0.03	0.76 ± 0.03	0.45 ± 0.02	0.55 ± 0.03	9.2 ± 1.3	29.3 ± 1.3	28.7 ± 1.3	35.0 ± 1.9

We further studied the isoprenoid flux ratio under nitrogen-limited conditions (Fig. 3b). The Rs265:pBBR-*ads* and Rs265_*∆phaB*:pBBR-*ads* strains, which rely exclusively on the MEP pathway for isoprenoid production, did not show any increase in the amorphadiene/biomass ratio when compared to nitrogen excess conditions (Fig. 3b). In contrast, the amorphadiene/biomass ratio for the strains that express only the MVA pathway was increased threefold when compared to this value under nitrogen excess conditions (Rs265-MVA_*∆dxr* and Rs265-MVA_*∆dxr∆phaB* strains harbouring pBBR-MVA-*ads* plasmid, Fig. 3). Interestingly, for the strains that express both pathways (Rs265:pBBR-MVA-*ads* and Rs265_*∆phaB*:pBBR-MVA-*ads* strains, Fig. 3b), there was only a minor increase of the MVA pathway capacity in the Rs265_*∆phaB* strain when compared to nitrogen excess conditions (Table 4). On the other hand, the MEP pathway capacity increased by 80% for the Rs265 strain (from 9.4 ± 0.3 to 16.9 ± 0.4 mg amorphadiene · g of biomass^−1^) and by 300% for the Rs265_*∆phaB* strain (from 9.2 ± 1.3 to 28.7 ± 1.3 mg amorphadiene · g of biomass^−1^). Thus, for the dual-pathway strain, the increase of the amorphadiene/biomass ratio under nitrogen limitation conditions is attributed to the endogenous MEP pathway (Table 4).

### Amorphadiene biosynthesis during resting cells conditions

Under nitrogen-limited conditions a short exponential growth phase occurred, and therefore a short growth-associated amorphadiene production phase could not be avoided. This resulted in non-linear growth and production kinetics, making it difficult to assess yields (mg amorphadiene · g glucose^−1^) and productivities (mg amorphadiene · L^−1^ · h^−1^). In order to focus exclusively on growth-uncoupled production, and to obtain linear kinetics, we decided to assess amorphadiene production during resting cell conditions in nitrogen-free medium. This cultivation setup simulates the production phase of a two-stage fermentation setup where growth and production are separated.

**Fig. 4:**
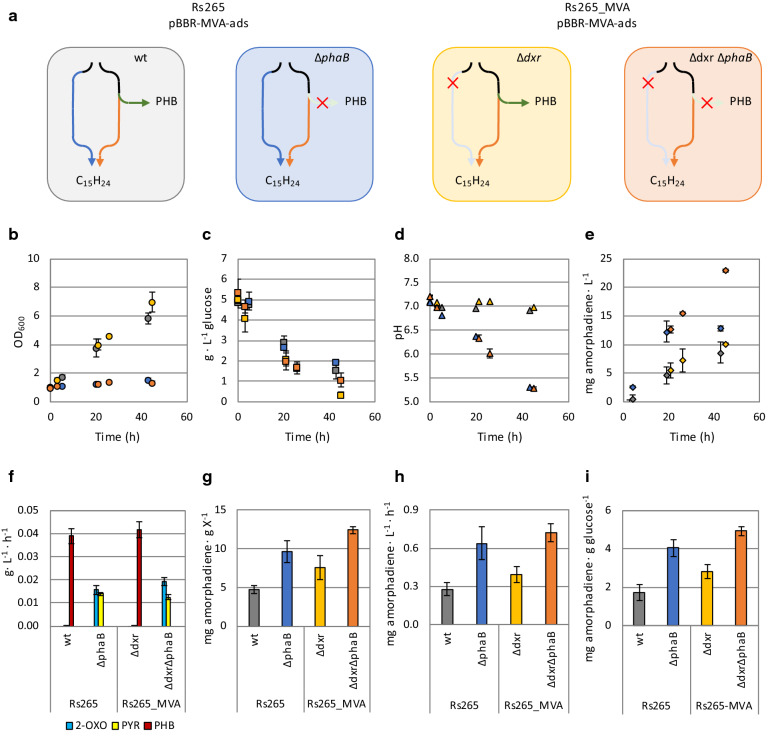
Growth-independent amorphadiene production. **a** Schematic overview of the strain tested. Blue arrows show the endogenous MEP pathway, while the orange ones the orthogonal MVA pathway. In green arrows, the PHB biosynthetic pathway is depicted. Red cross represent genes deletion of either *dxr* (MEP pathway) or *phaB* (PHB pathway) genes. C_15_H_24_ is the brute formula of the reporter molecule amorphadiene. **b**–**e** Monitoring of **b** OD_600_, **c** glucose concentration, **d** pH, **e** amorphadiene concentration. **f** volumetric productivities of PHB and organic acids (PYR: pyruvate; 2-OXO: 2-oxoglutarate). Determination of **g** amorphadiene/biomass ratios, **h** amorphadiene volumetric productivity and **i** amorphadiene yield on glucose

Since deletion of *phaB* and expression of the MVA pathway increased production during nitrogen limitation, we reasoned to assess the amorphadiene production levels in the presence of also an active MEP pathway. Therefore, we further cultivated the strains Rs265, Rs265_*∆phaB*, Rs265-MVA_*∆dxr* and Rs265-MVA_*∆dxr∆phaB* under resting cells condition. All these strains contained the pBBR-MVA-*ads* plasmid (Fig. 4a).

A linear increase was observed in the OD_600_ of the strains with a functional PHB biosynthetic pathway (Rs265 and Rs265-MVA_∆*dxr*, Fig. 4b). This trend is known to be associated with the accumulation of this storage compound [29], and it is associated with cell expansion rather than cell division. Accordingly, the corresponding ∆*phaB* strains (Rs265_∆*phaB* and Rs265-MVA_∆*dxr*∆*phaB*) did not show any increase in OD_600_. Glucose consumption (Fig. 4c, Additional file 1: Fig. S2), pH and amorphadiene concentrations (Fig. 4d, e) were followed over time. A decrease in the pH of the Rs265_∆*phaB* and Rs265-MVA_∆*dxr*∆*phaB* strains was observed (Fig. 4d), which can be explained by the secretion of organic acids upon prevention of PHB accumulation, mainly of pyruvate and 2-oxoglutarate (Fig. 4f).

Amorphadiene samples were collected over time from all the cultures (Fig. 4e), and yields and productivities were calculated for the first 24 h (Fig. 4g–i). The corresponding values for the Rs265 strain were the lowest among the tested strains. Deletion of *phaB* (Rs265_*∆phaB*) resulted in a twofold increase of the amorphadiene/biomass ratio (Fig. 4g, Additional file 1: Table S2), the volumetric productivity (Fig. 4h) and the yield on glucose (Fig. 4i) compared to the Rs265 strain. Also, inactivation of the endogenous MEP pathway in the Rs265-MVA_*∆dxr* strain resulted in an increase of those values compared to Rs265 strain (Fig. 4h, i). Hence, inactivation of either the PHB production pathway or the endogenous MEP pathway stimulates growth-independent production. Combined inactivation of the MEP and PHB production pathways (Rs265-MVA_*∆dxr∆phaB* strain) allowed this strain to reach the highest amorphadiene/biomass ratio, volumetric amorphadiene productivity (Fig. 4h) and yield on glucose (Fig. 4i). All these values were 2.5-fold higher in the Rs265-MVA_*∆dxr∆phaB* strain, compared to the Rs265 control strain. Hence, deletion of the endogenous MEP and PHB biosynthetic pathways resulted in the best metabolic setup for exploiting non-growing conditions for amorphadiene production.

## Discussion

Strain optimization for improved bioproduction often relies on strategies that couple production to microbial growth [19, 37]. Nevertheless, an emerging approach for metabolic engineering strategies is the one of dissociating production and growth [26]. Following this view, in this work we engineered the isoprenoid and the PHB biosynthetic pathways in *R. sphaeroides*. Therefore, we could demonstrate that isoprenoid biosynthesis, a typically growth-coupled type of metabolism in microorganisms, can be uncoupled from biomass production by means of rational metabolic engineering.

A previous work indicated that isoprenoid synthesis is strictly growth-coupled via the endogenous MEP pathway [29]. In the same study, the authors reported the first TRY parameters for this species, on a defined medium. They demonstrated that cultivation conditions can affect TRY values. By co-expressing both isoprenoid pathways, the yield of amorphadiene on glucose was highest under micro-aerobic conditions, reaching a value of about 0.005 mol · mol glucose^−1^ [29]. This value is still considerably low compared to the maximum theoretical yield that could be obtained from glucose (0.285 mol · mol glucose^−1^). Moreover, the maximal amorphadiene productivity was attained during exponential growth and reached 2 mg · L^−1^ · h^−1^. We applied nitrogen-limited conditions to a strain relying only on this isoprenoid pathway, but this did not result in increased amorphadiene/biomass ratio (Fig. 2b). We reasoned that targeting the storage compound (PHB) synthesis could increase the flux via the MEP pathway during this condition. We attempted to enhance the isoprenoid flux via this pathway because it presents the highest theoretical yield on glucose. Nevertheless, inactivation of the PHB synthetic pathway did not result in any improvement (Fig. 2b), thereby indicating that the endogenous MEP pathway is inhibited during non-growing conditions.

Differently from the MEP pathway, the MVA pathway is non-native in *R. sphaeroides*. Therefore, despite its lower theoretical yield, this heterologous pathway is expected to be less affected by endogenous regulation. Introduction of a heterologous MVA pathway was described to allow isoprenoid synthesis also during non-growing conditions [29]. Here, we confirmed that nitrogen-limited conditions increased the amorphadiene/biomass ratio in a strain relying exclusively on the MVA pathway (Fig. 2d). Moreover, inactivation of PHB synthesis by targeting *phaB* increased amorphadiene production when the MVA pathway was present (Fig. 2c, d). Because the current production of isoprenoids in *R. sphaeroides* is far from the maximum theoretical yield, the beneficial effect of MVA pathway expression on the overall isoprenoid flux compensated its lower theoretical yield. In order to calculate titers, rates and yields (TRY) of growth-independent amorphadiene synthesis, we performed cultivation under resting cells condition (Fig. 4). The experimental data confirmed that exclusive isoprenoid flux via the MVA pathway combined with inactivation of PHB synthesis results in maximal TRY values (Fig. 4e, g–i).

^13^C metabolic flux ratio analysis was performed to understand the interaction between the two isoprenoid pathways during nitrogen limitation. The analysis indicated that in the dual-pathway strain the endogenous MEP pathway capacity is substantially enhanced during nitrogen limitation (Fig. 3b). Therefore, presence of the MVA pathway helps in deregulating the endogenous MEP pathway during nitrogen limitation.

Despite the increase in flux, only a small part of the carbon that originally went to PHB production could be redirected to amorphadiene. Organic acids—especially pyruvate and 2-oxoglutarate—were excreted instead. The accumulation of these two organic acids indicates that their downstream reactions, catalysed by the pyruvate dehydrogenase and 2-oxoglutarate dehydrogenase complexes, respectively, were inhibited by the inability to produce PHB. These reactions require input of free CoA (Fig. 1), the co-enzyme released by the conversion of AA-CoA into PHB. We therefore speculate that knocking out either *phaB* or *phaC1* and *phaC2* decreased the availability of free CoA, that the capacity of the mevalonate pathway was insufficient to remedy this, and that this low CoA availability resulted in the accumulation of both pyruvate and 2-oxoglutarate. Possibly, secretion of 3HB after deletion of *phaC1* and *phaC2* allowed to release free CoA from the intermediate 3HB-CoA.

PHB synthesis results in [38]: (i) carbon storage, (ii) regeneration of NADP^+^ and (iii) regeneration of free CoA for cellular homeostasis. Knocking out the ability to produce PHB should therefore increase the availability of precursors (AA-CoA) and NADPH for the MVA pathway. The relatively small increase in flux through the MVA pathway indicated that this pathway benefited from the increased amounts of AA-CoA and NADPH, but its capacity is limiting isoprenoid production. One of the rate-determining enzymes of the heterologous pathway is *HMG*-*CoA reductase*, already described as crucial enzyme for enhancing isoprenoid flux [15, 39]. We speculate that improving the catalytic efficiency of this enzyme might allow the MVA pathway to benefit from the higher availability of NADPH and AA-CoA. Such an improvement could allow to increase carbon flux towards isoprenoids, while reducing by-products secretion. Therefore, rational engineering strategies can build upon the findings of this work for further improving growth-independent isoprenoid biosynthesis in *R. sphaeroides*.

## Conclusions

In this work, we assessed the contribution of the MEP and MVA pathways to amorphadiene biosynthesis under different culturing conditions. We confirmed that application of the heterologous MVA pathway holds potential for growth-independent production. In a dual-pathway strain, enhancement of the endogenous MEP pathway capacity was confirmed during nitrogen-limited conditions via ^13^C metabolic flux ratio analysis.

Nevertheless, isoprenoid synthesis during resting cells condition was limited by the presence of an active endogenous MEP pathway. On the other hand, exclusive isoprenoid flux via the MVA pathway increased amorphadiene synthesis during this condition. Additionally, prevention of PHB synthesis via *phaB* resulted in the highest TRY values for growth-independent amorphadiene production.

Ultimately, this work proposed a metabolic engineering design for increasing growth-independent isoprenoid biosynthesis in *R. sphaeroides*, while providing novel insights about the interaction occurring between the two isoprenoid pathways.

## Supplementary information

**Additional file 1.** Additional figures and tables.

## Data Availability

The dataset supporting the conclusions of this article are included within the article and in the additional file.

## Abbreviations

PHB: Poly-β-hydroxybutyrate
MEP: 2-Methyl-d-erythritol 4-phosphat
MVA: Mevalonate
CRISPR–Cas: Clustered regularly inter‑spaced short palindromic repeats–CRISPR-associated proteins
PYR: Pyruvate
CoA: Coenzyme-A
NADPH: Dihydronicotinamide-adenine dinucleotide phosphate
Ac-CoA: Acetyl-CoA
AA-CoA: Acetoacetyl-CoA
HMG-CoA: Hydroxymethylglutaryl-CoA
3-HB-CoA: 3-Hydroxybutyryl-CoA
3HB: 3-Hydroxybutyrate
2-OXO: 2-Oxoglutarate
*phaB*: Acetoacetylreductase (gene)
*dxr*: 1-Deoxy-d-xylulose 5-phosphate reductoisomerase (gene)
*ads*: Amorphadiene synthase (gene)
