# Supplementary material for: Growth-uncoupled isoprenoid synthesis in Rhodobacter sphaeroides
Source: Biotechnol Biofuels. 2020 Jul 13;13:123. doi: 10.1186/s13068-020-01765-1 (PMC7359475; doi:10.1186/s13068-020-01765-1)
Supplement: Supplementary file 1 — Additional file 1. Additional figures and tables. [file 13068_2020_1765_MOESM1_ESM.docx]

Growth-uncoupled isoprenoid synthesis in *Rhodobacter sphaeroides*

Enrico Orsi^1,6*^, Ioannis Mougiakos^2,7*^, Wilbert Post^1,2^, Jules Beekwilder^3^, Marco Dompè^4^, Gerrit Eggink^1,5^, John van der Oost^2^, Servé W. M. Kengen^2^, Ruud A. Weusthuis^1,**^

^1^Bioprocess Engineering, Wageningen University, Droevendaalsesteeg 1, 6708 PB Wageningen, The Netherlands

^2^Laboratory of Microbiology, Wageningen University, Stippeneng 4, 6708 WE Wageningen, The Netherlands

^3^Wageningen Plant Research, 6700AA Wageningen, The Netherlands

^4^Physical Chemistry and Soft Matter, Wageningen University, Stippeneng 4, 6708 WE Wageningen, The Netherlands

^5^Wageningen Food & Biobased Research, 6708WG Wageningen, The Netherlands

^6^Present address: Systems and Synthetic Metabolism Group, Max Planck Institute of Molecular Plant Physiology, Am Mühlenberg 1, 14476 Potsdam, Germany.^7^Present address: Helmholtz Institute for RNA-based Infection Research (HIRI), Helmholtz-Centre for Infection Research (HZI), 97080 Würzburg, Germany.

^*^contributed equally

^**^corresponding author, [ruud.weusthuis@wur.nl](mailto:ruud.weusthuis@wur.nl)

# Additional information

**Table S1.** Primers used in this study.

| **Scope** | **Assembly fragments** | **Primer #** | **Sequence** | **Description** |
| --- | --- | --- | --- | --- |
| *phaB* knock-out | #1 | P383 | **GACCTACAAATGGTCCGTCG**GTTTTAGAGCTAGAAATAGCAAGTTAAAATAAGGCTAGTC | Forward primer for PCR amplification of the sgRNA scaffold and the *hcas9* gene from the pBBR_Cas9NT vector, with the *phaB-*targeting spacer 1 sequence (bold) as overhung |
|  |  | P385 | **CGGGCAGGCGAACTATTCGG**GTTTTAGAGCTAGAAATAGCAAGTTAAAATAAGGCTAGTC | Forward primer for PCR amplification of the sgRNA scaffold and the *hcas9* gene from the pBBR_Cas9NT vector, with the *phaB-*targeting spacer 2 sequence (bold) as overhung |
|  |  | P387 | **CAATCTCCTCCGGTTCGCCG**GTTTTAGAGCTAGAAATAGCAAGTTAAAATAAGGCTAGTC | Forward primer for PCR amplification of the sgRNA scaffold and the *hcas9* gene from the pBBR_Cas9NT vector, with the *phaB-*targeting spacer 3 sequence (bold) as overhung |
|  |  | P301 | CCATGTCGGCAGAATGCTTAATG | Reverse primer for PCR amplification of the sgRNA scaffold and the *hcas9* gene from the pBBR_Cas9NT vector. |
|  | #2 | P302 | CATTAAGCATTCTGCCGACATGG | Forward primer for PCR amplification of the pBBR_Cas9NT backbone (reverse complement of primer P301). |
|  |  | P303 | GCCTGAATGGCGAATGGAAATTGTAA | Reverse primer for PCR amplification of the pBBR_Cas9NT backbone |
|  | #3 | P382 | **TTACAATTTCCATTCGCCATTCAGGC**GAGCCTCTGTCTCGC | Forward primer for PCR amplification of the *phaB* homologous recombination flank 1 from the *R. sphaeroides* genome. The overhang (bold) is reverse complement of primer P303. |
|  |  | P381 | GGATATTTGAAGGAGGGATC**GGATCTTCTCATCCTGAAAACCA** | Reverse primer for PCR amplification of the *phaB* homologous recombination flank 1 from the *R. sphaeroides* genome. The overhang is reverse complement of the non-overhung part of primer P380, while the whole primer is reverse complement of primer P380. |
|  | #4 | P380 | **TTTTCAGGATGAGAAGATCC**GATCCCTCCTTCAAATATCCGC | Forward primer for PCR amplification of the *phaB* homologous recombination flank 2 from the *R. sphaeroides* genome. The overhang is reverse complement of the non-overhung part of primer P381, while the whole primer is reverse complement of primer P381. |
|  |  | P379 | **TAATAGCGAAGAGGCCCGCAC**ATGTCCAGCTGGGCGAC | Reverse primer for PCR amplification of the *phaB* homologous recombination flank 2 from the *R. sphaeroides* genome. The overhang (bold) is reverse complement of primer P304. |
|  | #5 | P304 | GTGCGGGCCTCTTCGCTATTA | Forward primer for PCR amplification of the pBBR_Cas9NT backbone region between the *phaB* homologous recombination flank 2 and the J95023 promoter of the sgRNA module |
|  |  | P384 | **CGACGGACCATTTGTAGGTC**AACCAGCGATCCCGTCCG | Reverse primer for PCR amplification of the pBBR_Cas9NT backbone region between the *phaB* homologous recombination flank 2 and the J95023 promoter of the sgRNA module, with the reverse complement of the *phaB-*targeting spacer 1 sequence (bold) as overhung (the overhung is reverse complement of the overhung of primer P383) |
|  |  | P386 | **CCGAATAGTTCGCCTGCCCG**AACCAGCGATCCCGTCCG | Reverse primer for PCR amplification of the pBBR_Cas9NT backbone region between the *phaB* homologous recombination flank 2 and the J95023 promoter of the sgRNA module, with the reverse complement of the *phaB-*targeting spacer 2 sequence (bold) as overhung (the overhung is reverse complement of the overhung of primer P385) |
|  |  | P388 | **CGGCGAACCGGAGGAGATTG**AACCAGCGATCCCGTCCG | Reverse primer for PCR amplification of the pBBR_Cas9NT backbone region between the *phaB* homologous recombination flank 2 and the J95023 promoter of the sgRNA module, with the reverse complement of the *phaB-*targeting spacer 2 sequence (bold) as overhung (the overhung is reverse complement of the overhung of primer P387) |
| sequencing | - | P409 | AGCATCAACCAGGTCTGCGG | Genome specific primer for screening of *R. sphaeroides phaB* deletion mutants |
|  |  | P410 | ACAACGGCATCCCGACGA | Genome specific primer for screening of *R. sphaeroides phaB* deletion mutants |

**Table S2.** Yield of amorphadiene on biomass calculated during resting cells condition in nitrogen free medium.

| **Plasmid** | **Cultivation condition** | **Genomic background** | **Amorphadiene on biomass, *Y_P/X_* (mg ∙ g^-1^)** | |
| --- | --- | --- | --- | --- |
|  |  |  | **Value** | **St. dev.** |
| pBBR-MVA-ads | Resting cell | *wt* | 4.7 | 0.5 |
|  |  | *ΔphaB* | 9.6 | 1.4 |
|  |  | *Δdxr* | 7.6 | 1.6 |
|  |  | *ΔdxrΔphaB* | 12.4 | 0.5 |

# Figures

**Fig. S1. NMR analysis of the spent medium for 3-hydroxybutyrate (3HB) determination.** The presence of the two hydrogen a,b close to the carboxylic groups was detected at 2.34 and 2.44 (both multiplets). The hydrogen c close to the hydroxyl group has been identified at 4.10 (multiplet). The three hydrogen belonging to the methyl group have been identified at 1.10 (doublet). The ratio among the hydrogen a:c:d is 1:1:3 as expected from the structure of the molecule. The hydrogen b at 2.44 is overlapping with the signal from other groups so it cannot be used for quantification. The results are in line with literature data.

**Figure S2. Glucose consumption rates during resting cells conditions.** All strains harbored the pBBR-MVA-*ads* plasmid. Colors refer to different strains tested: gray (wild-type), blue (∆*phaB*), yellow (∆*dxr*), red (∆*dxr*∆*phaB*).
